# Suicide and Attempted Suicide in Poland before and during the COVID-19 Pandemic between 2019 and 2021

**DOI:** 10.3390/ijerph19158968

**Published:** 2022-07-23

**Authors:** Jacek Stańdo, Adam Czabański, Żywilla Fechner, Ewa Baum, Karl Andriessen, Karolina Krysińska

**Affiliations:** 1 Centre of Mathematics and Physics, Lodz University of Technology, 90-924 Lodz, Poland; 2 Department of Social Sciences, Jacob of Paradies University, 66-400 Gorzów Wielkopolski, Poland; aczabanski@interia.pl; 3 Institute of Mathematics, Lodz University of Technology, al. Politechniki 8, 93-590 Łódź, Poland; zywilla.fechner@p.lodz.pl; 4 Department of Social Sciences and the Humanities, Poznan University of Medical Sciences, 60-806 Poznan, Poland; ebaum@ump.edu.pl; 5 Melbourne School of Population and Global Health, The University of Melbourne, Melbourne, VIC 3010, Australia; karl.andriessen@unimelb.edu.au (K.A.); karolina.krysinska@unimelb.edu.au (K.K.)

**Keywords:** suicide, COVID-19

## Abstract

The COVID-19 pandemic is related to changes in rates of suicide and suicide attempts in many countries, and some differences have been observed regarding the prevalence of suicidal behaviours in different age and gender groups. The aim of this study is to analyse the number of suicides and suicide attempts per 100,000 people between 2019 and 2021 in Poland. Using police and government data on suicide and suicide attempts in Poland, three age categories were investigated: 13–24 years old, 25–65 years old, and above 65 years old, and the analysis encompassed the whole population and the populations of men and women separately. Study results indicated an increase in suicide attempts in the two younger age categories (aged 7–24 years and 25–65 years) between 2021 and 2019–2020. There was an increase in suicide among women in all age categories during the study period, whilst no increase was observed in suicide in men in any age group. The differences in the prevalence of suicide and attempted suicide in Poland during the COVID-19 pandemic in different age and gender groups indicate the need for tailored suicide prevention activities.

## 1. Introduction

Over the years, research has confirmed that the risk of attempted suicide decreases with age [1]. Pan-European studies have shown that only 9% of the suicide attempts recorded in hospitals have been made by elderly people (65+), whilst adolescents (14–15 years) account for 50% of all those who have attempted suicide [2]. Two to four attempts per suicide occur among elderly persons [3]. On the other hand, adolescents and young adults make 100 to 200 suicide attempts per suicide [4]. Similar trends have been reported by Hołyst in reference to the WHO Suicide Prevention Multisite Intervention Study on Suicidal Behaviours (WHO/SUPRE-MISS). The share of older people who had attempted in suicide was 1% [5].

Some studies provide information that 85% of elderly people take their own life already in the first attempt [6]. The high mortality of suicide behaviour by older people (65+) is at least partly due to the fact that their actions are carefully planned and less impulsive than those of younger people. Moreover [5], seniors choose more lethal methods (they may have experience in this area due to their age) and use alcohol less frequently in their attempts than younger people [5]. It should also be mentioned that the general determination in wanting one’s own death via the actual suicidal behaviour is stronger among the elderly than among younger people.

In the group of people of working age (25–65 years), suicide attempts occur 10 or even 20 times more often than suicide [7,8]. Since the fall of the communist regimes in 1990, the number of suicides among people of working age has gradually decreased. This trend continues in the following years of the 21st century. In each of the countries surveyed, men of working age commit suicide many times more often than women [8]. In the United States, between 2000 and 2017, there was a 40% increase in the number of suicides among people of working age. In the period before the COVID-19 pandemic, the severity of suicide among men of working age was 27.4/100,000, and among women of working age 7.7/100,000 [9]

The emergence of the COVID-19 pandemic raised questions among researchers about its impact on the psychosocial processes of suicides. An increase in the number of suicides was expected [10]. Studies conducted in various countries have shown that the first months of the COVID-19 pandemic did not bring a significant increase in the number of suicides, and the psychosocial processes of suicides were even [11,12,13,14,15]. However, it was noted that the COVID-19 pandemic has had a differentiated impact on men and women in terms of the psychosocial processes of suicide observed among them [16].

However, the inconveniences and threats caused by the COVID-19 pandemic, which was first officially recorded in Poland on 4 March 2020, did not seem to trigger a higher number of suicide attempts by elderly people. This is based on official government statistics and is discussed later in this article.

As Japanese research shows an increase in the number of female suicide attempts and suicides during the COVID-19 pandemic in Japan (increased unemployment among women and an increase in incidents of violence against them), a similar trend can be expected in Poland [17].

The aim of the research was to determine the dynamics of suicide in Poland during the COVID-19 epidemic in relation to gender; the aim of the research was also to determine the dynamics of suicides in Poland during the COVID-19 epidemic in particular age groups, especially adolescents and seniors. Study results indicate an increase in suicide attempts in the two younger age categories (7–24 years and 25–65 years) between 2021 and 2019–2020. There was an increase in suicide among women in all age categories during the study period, whilst no increase was observed in suicide in men in any age group.

## 2. Materials and Methods

The data on the number of suicides and attempted suicides was provided by police statistics. These are published on: [18] 

We used this data to show that in 2021 one can observe the increase in the number of suicides (i.e., deaths by suicide) and suicide attempts per 100,000 people with respect to 2019 and 2020. Our aim is to investigate three age categories: first, people between 13 and 24 years old; second, people between 25 and 65 years old; and third, people above 65 years old. We investigated the whole population and the populations of men and women separately. We applied the sequence of tests for proportion for all categories with Bonferroni corrections for multiple testing. First, we considered the percentage rate of suicides or attempted suicides and second, we applied the test for two proportions. We used the prop.test() function in R. We formulated the conclusions in terms of the number of suicides per 100,000 people. The specific hypothesis are discussed below.

**Figure 1:**
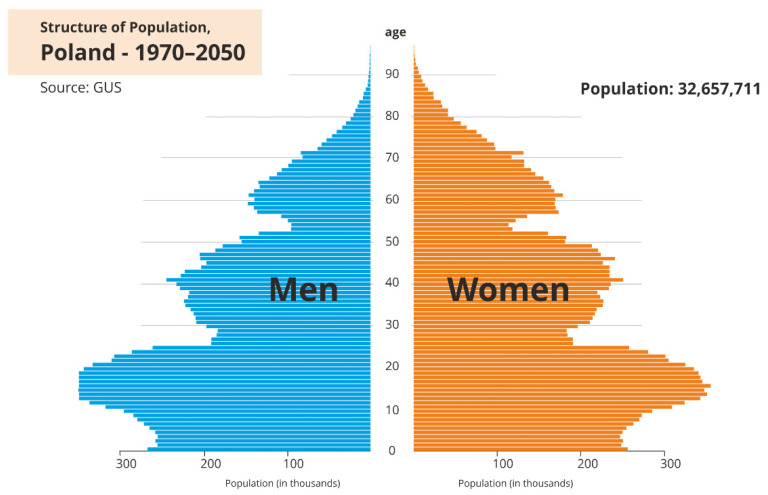
Population size in Poland [19].

In the statistical analysis of data, it is necessary to take into account the size of the appropriate age categories. More precisely, the population of people in Poland in 2021 aged 13 to 24 was approximately 4.556 million, the population of people aged 25 to 64 was 20.714 million, whilst the population of people over 65 was 7.417 million people. This means that the size of the first age category is more than four times smaller than the size of the second age category, hence the need to consider the size of the appropriate age categories and to use proportions. Data on the number of people are presented in the form of a pyramid which depicts the structure of the population in Poland (Figure 1).

**Figure 2:**
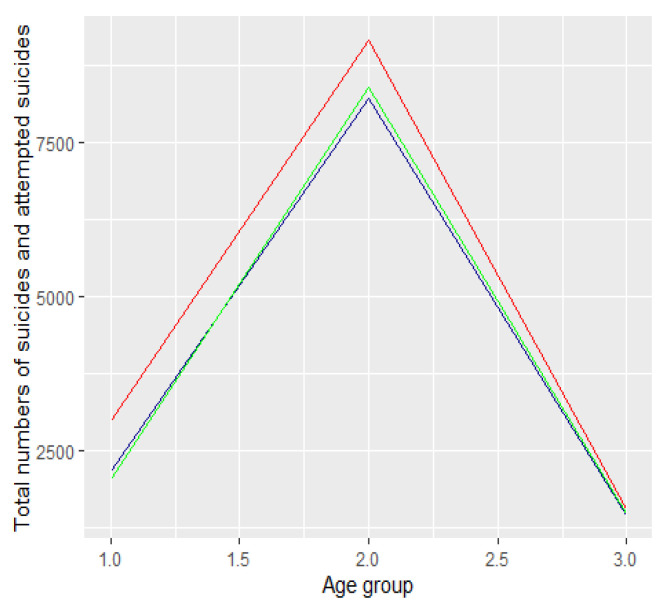
Absolute number of suicide attempts in Poland between 2019 and 2021.

**Figure 3:**
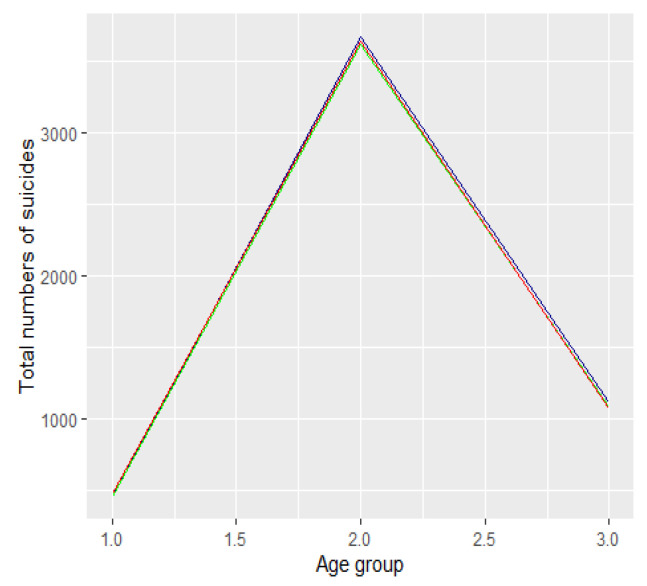
Absolute number of suicides in Poland between 2019 and 2021.

The absolute number of suicide attempts [resp. suicides] between 2019 and 2021 by age category is presented in Figure 2 [resp. Figure 3]. The dark blue colour refers to 2019 data, the green refers to 2020, and the red to 2021.

**Figure 4:**
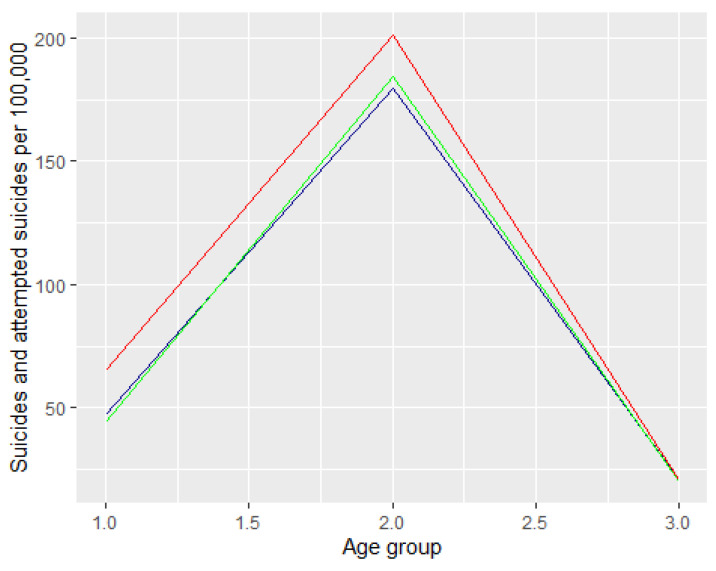
The number of suicide attempts per 100,000 in Poland between 2019 and 2021.

**Figure 5:**
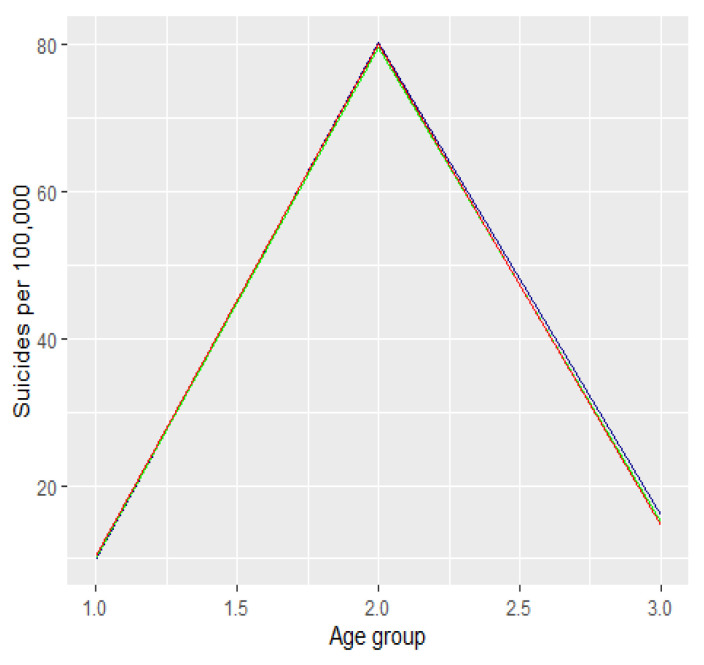
The number of suicides per 100,000 in Poland between 2019 and 2021.

We looked at the number of attempted suicides in relation to the size of each age group. To do so, we determined the size of each age group in the period between 2019 and 2021. We used statistical data published on https://stat.gov.pl/obszary-tematyczne/ludnosc/ludnosc/ludnosc-piramida/ (accessed on 18 December 2021 month year)). Next we expressed this rate in terms of common metrics, namely the number of attempted suicides per 100,000 people. The graph below shows the number of suicide attempts per 100,000 people among men and women expressed in the respective age groups. The dark blue colour refers to 2019 data, the green refers to 2020, and the red to 2021. The first graph (Figure 4) refers to the percentage of the number of suicide attempts per 100,000 in the relevant age groups. The second graph (Figure 5) refers to the number of suicide attempts per 100,000.

## 3. Results

We divided the groups into three age categories. In the first category we included people aged from 13 to 24 years, i.e., groups 2 and 3 (Figure 1 and Figure 2). In the second category we included people aged 25 to 64, i.e., age groups from categories 4 to 11. The third category included people aged 65 years and over, i.e., age groups 12 to 16. We compared the number of suicide attempts between 2019 and 2021 in each of the three categories. We conduced analyses for all suicide attempts as well as suicides. In both cases, we used proportion tests for two or multiple populations with Bonferroni correction for multiple testing. We used the RStudio environment for the analyses (RStudio 2021.09.01, R Version 4.1.2). We assumed a significance level of 0.05. Using the Bonferroni correction we compared the p-values with the significant levels 0.05/m, where m denotes the number of tests conducted in the multiple testing. For the description of the statistical tests and the form of statistics see, e.g., [20].

Let us assume that p(19), p(20) and p(21) signify the percentage of all suicide attempts in first category in 2019, 2020 and 2021, respectively. Let us test the following hypothesis:

**Hypothesis** **0** **(H0).** *p*(*year*1) = *p*(*year*2).

against the alternative hypothesis:

**Hypothesis** **1** **(H1).** *p*(*year*1) < *p*(*year*2). for all the possible pairs: year1, year2 in the date range from 2019 to 2021. We conducted a corresponding analysis for the second and third age categories. We conducted the analysis for all suicide attempts as well as suicides.

### 3.1. Summary of All Suicide Attempts

First we show that there is an increase in the number of attempted suicides from 2019 to 2021 and from 2020 to 2021 in the first and second category. There is no statistically significant increase in the third category. Moreover, no increase is observed in any category in the number of suicides per 100,000. The corresponding p-values are summarized in the Table 1.

First category: there was an increase in the number of attempted suicides per 100,000:

1. in 2021 in relation to 2019
2. in 2021 in relation to 2020

No statistically significant changes were observed in other years.

Second category: there was an increase in the number of attempted suicides per 100,000:

3. in 2021 in relation to 2019
4. in 2021 in relation to 2020

No statistically significant changes were observed in other years.

Third category: there were statistically significant changes in the number of attempted suicides per 100,000 in the specified period.

### 3.2. Summary for Suicide Attempts Ending in Death

Results of proportion tests for suicides are reported in Table 2.

Third category: There were statistically significant changes in the number of attempted suicides per 100,000 in the specified period.

### 3.3. Gender and Suicide Attempts

We now analyze suicide attempts in the age categories defined above, broken down by gender. We only considered the cases where the gender had been identified for the analyses. We considered the number of female suicide attempts per 100,000 inhabitants in the respective groups, as presented in Table 3.

**Figure 6:**
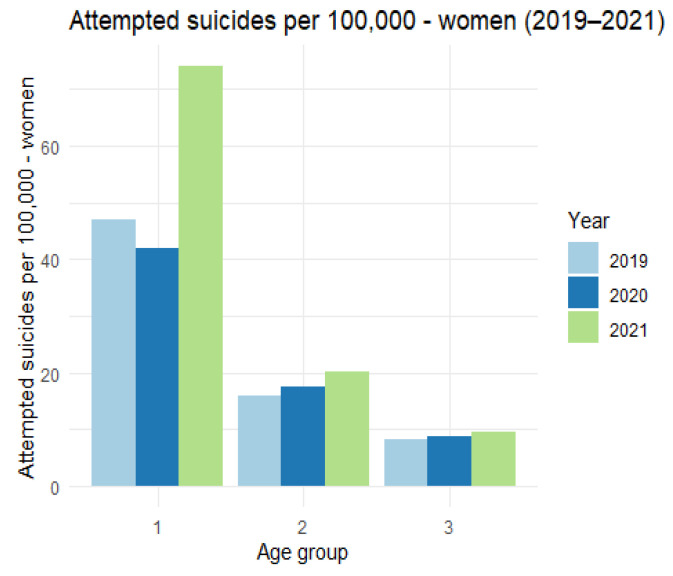
The number of attempted suicides per 100,000 women (2019–2021).

The above data can be presented graphically (Figure 6).

**Figure 7:**
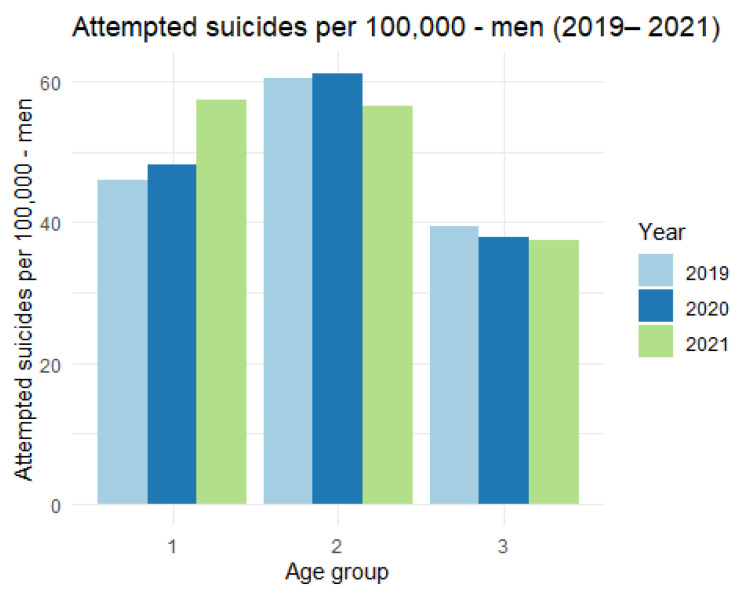
The number of attempted suicides per 100,000 men (2019–2021).

There is a clear increase in the number of suicide attempts among women per 100,000 inhabitants in the first age category. In the remaining categories, the differences are much smaller. We now turn to the analysis of the number of suicide attempts among men per 100,000 inhabitants, presenting the relevant data in Table 4 and Figure 7.

In this case, we also observe an increase in the number of suicide attempts among men per 100,000 inhabitants in the first category, but it is not as significant as in the women’s group. 

**Figure 8:**
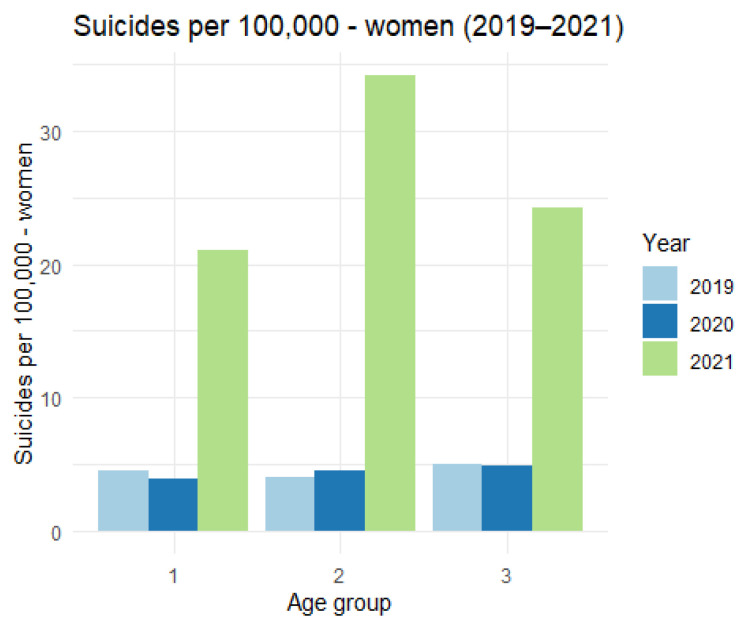
The number of suicides per 100,000—women (2019–2021).

We now turn to the analysis of suicides in the examined age categories, broken down by gender. The data on the group of women are as follows (Table 5 and Figure 8.)

The figure shows a very significant increase in the number of suicides per 100,000 inhabitants among women in all age categories.

**Figure 9:**
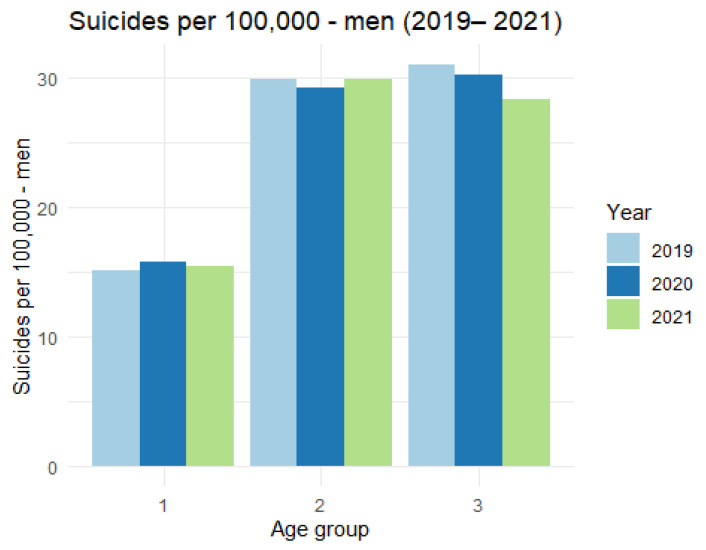
The number of suicides per 100,000 men.

The data on suicides among men are presented in Table 6 and Figure 9.

The analysis of the figure shows that there are no significant differences in the number of suicides per 100,000 inhabitants in the analyzed age categories. 

It is worth noting that female suicides account for approximately 20% of all suicides. The number of women and men in the first two categories is comparable, whilst in the third category, i.e., people over 65, the size of the female population is approximately 60% of the total population of the third category.

The above considerations were supported by statistical tests. The methodology was analogous to that used and described for the population with no gender division. A summary of the relevant p-values is provided below (see Table 7 and Table 8 for details). “Yes” means there was an increase in 2021, “No” means there is no basis to claim there was an increase in 2021.

It can be seen that an increase in the number of suicide attempts occurs in the first and second age categories among women each year. We do not observe such an increase in the third category among women. In the case of men, the increase is significant in the first age group and there is no basis to conclude that the increase occurs in the second and third age categories.

In the case of female suicides, an increase is observed in all age categories and all years, whilst there is no basis for an increase in any age category in the case of male suicides.

## 4. Discussion

There was an increase in suicide attempts in 2021 compared to 2019–2020 in the younger age categories I (7–24 years) and II (25–65 years). The pandemic situation impacted adolescents differently than adults (working age). Adolescents were particularly adversely affected by forced isolation and participation in distance (online) learning. Working-age adults, on the other hand, feared losing their jobs, rising loan and mortgage rates as well as higher cost of living. However, Poland managed to keep the unemployment rate at a low level. At the end of January 2019, the unemployment rate in Poland was 6.1% (1023.1 thousand unemployed) (Central Statistical Office: stat.gov.pl/obszary-tematyczne/rynek-pracy/unemployment-registered/unemployed-registered-unemployment-status-w-koncu-stycznia-2019-r-,2,78html (accessed on 28 March 2022)). Moreover, despite the COVID-19 pandemic, unemployment in Poland decreased. At the end of December 2021, the unemployment rate in Poland was 5.4%, i.e., 895,700 people (Ministry of Family and Social Policy: [21]. Several million jobs were saved, which translated into keeping suicide rates in Poland at the same level despite some increase in suicide attempts. Otherwise, if the unemployment rate had grown, there would have been a rise in suicides in Poland as well. R. Łukasiewicz wrote about high correlations between unemployment and suicides in Polish society [22]. This seems to be confirmed by Japanese research. In Japan, during the COVID-19 pandemic unemployment increased, affecting mostly women. In fact, unemployment, especially among women, and the recording of more violent behaviour during forced isolation in homes, was the main factor in the increase in suicides in Japan [23,24].

Pandemic daily life contributed to lower moods among Poles. This is confirmed by research conducted on a sample of 595 adults (80% women and 20% men) [25]. Earlier Polish studies also pointed to such a trend [26]. A review of international studies also confirmed the impact of the COVID-19 pandemic on the risks to mental well-being [27]. Interestingly, research findings show that women experience higher levels of tension and greater intensity of negative emotions [28,29,30,31]. Still, psychological costs do not translate into a significant increase in suicide rates. This is the case in the Polish population, whereas a Japanese study from September 2020 found that women experienced a higher suicide risk from COVID-19 than men, and this was approximately 20–30% higher than in previous years [32]. Studies have also found that people at younger ages (up to 22 years) experience higher levels of tension and less positive feelings than older people [28,33]. These reports may prove crucial in explaining the phenomenon of suicidal behaviour among young people in Poland, manifested by an increase in the number of suicide attempts and some stabilisation in the number of suicides. Individuals in late adolescence (school and university students), due to their age, may have fewer coping skills to handle the stress associated with the COVID-19 pandemic than older individuals. In their study of 2020, Wang and his colleagues determined that students exhibited higher levels of anxiety and depression [31,32,33,34,35,36,37]. During the COVID-19 pandemic, the medical care system focused on combating the effects, whilst other conditions were not always given the same attention. Hospitals reduced admissions for non-covid reasons. One should note that suicidology studies have shown that hospitalisation increases the risk of seniors engaging in suicidal behaviour. For many older people, hospitalisation is a highly traumatic experience, hence the fall in the overall number of non-covid hospitalisations among the elderly may also have contributed to some extent to stabilising the dynamics of suicidal behaviour. This might have been brought about by the numerous grassroots voluntary initiatives that emerged after the first and second waves of the COVID-19 pandemic, which provided assistance to the elderly, especially those who were lonely and lacked support from their family and friends.

## 5. Conclusions

Suicide is the result of many causes and risk factors, ranging from psychological and social to environmental and cultural to biological. The COVID-19 pandemic, in turn, is a state of emergency. It can be compared to a state of war, which, being an emergency, triggers in people a greater sense of solidarity with society than in times of peace. In such circumstances, individual problems are pushed into the background, whilst the most important thing is mobilisation and the struggle for survival as well as coping with the chronic stress that accompanies people during the pandemic period. Probably also for this reason, the suicide rates in Poland have become stable in all the analysed age categories, whilst the rates of suicide attempts have increased among people of pre-working and working age. This is a pessimistic prognosis, because after the end of the COVID-19 pandemic in Poland, there will also be a gradual increase in suicide rates.

## Figures and Tables

**Table 1:** Summary of proportion test—attempted suicides.

Year		Category I		Category II		Category III	
year1	year2	*p*-Value	*p*(year1) < *p*(year2)	*p*-Value	*p*(year1) < *p*(year2)	*p*-Value	*p*(year1) < *p*(year2)
2019	2020	9.77 × 10^−1^	NO	2.94 × 10^−2^	NO	7.34 × 10^−1^	NO
2019	2021	3.18 × 10^−31^	YES	5.81 × 10^−25^	YES	5.29 × 10^−1^	NO
2020	2019	2.30 × 10^−2^	NO	9.71 × 10^−1^	NO	2.66 × 10^−1^	NO
2020	2021	5.77 × 10^−2^	YES	3.44 × 10^−17^	YES	2.93 × 10^−1^	NO
2021	2019	1.00	NO	1.00	NO	4.71 × 10^−1^	NO
2021	2020	1.00	NO	1.00	NO	7.07 × 10^−1^	NO

**Table 2:** Summary of proportion test—suicides.

Year		Category I		Category II		Category III	
year1	year2	*p*-Value	*p*(year1) < *p*(year2)	*p*-Value	*p*(year1) < *p*(year2)	*p*-Value	*p*(year1) < *p*(year2)
2019	2020	0.458	NO	0.605	NO	0.918	NO
2019	2021	0.291	NO	0.055	NO	0.990	NO
2020	2019	0.542	NO	0.395	NO	0.082	NO
2020	2021	0.340	NO	0.030	NO	0.818	NO
2021	2019	0.709	NO	0.945	NO	0.010	NO
2021	2020	0.660	NO	0.970	NO	0.182	NO

**Table 3:** The number of suicide attempts among women per 100,000 inhabitants in 2019–2021.

Category	2019	2020	2021
1	47.07	41.94	74.02
2	15.94	17.52	20.24
3	8.2	8.78	9.56

**Table 4:** The number of attempted suicides per 100,000 men (2019–2021).

Category	2019	2020	2021
1	45.95	48.26	57.38
2	60.51	61.08	56.45
3	39.42	37.97	37.41

**Table 5:** Number of suicides per 100,000 women (2019–2021).

Category	2019	2020	2021
1	4.47	3.88	21.1
2	4.05	4.47	34.13
3	4.99	4.85	24.22

**Table 6:** Number of suicides per 100,000—men (2019–2021).

Category	2019	2020	2021
1	15.09	15.83	15.47
2	29.92	29.23	29.95
3	31.04	30.24	28.42

**Table 7:** Summary of test results for proportions—suicide attempts.

Attempted Suicides					
Sex	Category	year19 < year21		year20 < year21	
		*p*-Value	Conclusion	*p*-Value	Conclusion
Female	1	3.63 × 10^−31^	YES	3.53 × 10^−46^	YES
	2	6.22 × 10^−14^	YES	4.23 × 10^−7^	YES
	3	1.80 × 10^−2^	NO	1.22 × 10^−1^	NO
Male	1	2.98 × 10^−8^	YES	4.86 × 10^−^6	YES
	2	1.00	NO	1.00	NO
	3	8.89 × 10^−1^	NO	6.28 × 10^−1^	NO

**Table 8:** Summary of test results for proportions—deaths by suicide.

Suicides					
Sex	Category	year19 < year21		year20 < year21	
		*p*-Value	Conclusion	*p*-Value	Conclusion
Female	1	1.57 × 10^−54^	YES	5.52 × 10^−60^	YES
	2	0.00	YES	0.00	YES
	3	1.71 × 10^−121^	YES	2.08 × 10^−123^	YES
Male	1	3.84 × 10^−1^	NO	5.75 × 10^−1^	NO
	2	4.91 × 10^−1^	NO	1.06 × 10^−1^	NO
	3	9.66 × 10^−1^	NO	8.98 × 10^−1^	NO

## Data Availability

All data sources are cited directly in the paper.
